# Association Between the Naples Prognostic Score and In-Hospital Mortality in Patients with Type 2 Cardiorenal Syndrome: A Retrospective Cohort Study

**DOI:** 10.3390/diagnostics16193185

**Published:** 2026-09-30

**Authors:** Eren Eynel, Emre Eynel, Yusuf Can

**Affiliations:** 1 Department of Nephrology, Gazi Yaşargil Training and Research Hospital, 21070 Diyarbakır, Turkey; 2 Department of Cardiology, Sakarya University Training and Research Hospital, 54100 Sakarya, Turkey; emreeynel@gmail.com (E.E.); yusufcan@sakarya.edu.tr (Y.C.)

**Keywords:** cardiorenal syndrome, Naples Prognostic Score, inflammation, in-hospital mortality, risk stratification

## Abstract

**Objective:** This study evaluated the association between admission Naples Prognostic Score (NPS) and in-hospital mortality in patients with clinical features consistent with type 2 cardiorenal syndrome (CRS-2). **Methods:** This retrospective, single-center study included 101 adults hospitalized for decompensated heart failure at Sakarya University Training and Research Hospital between June 2021 and August 2024. CRS-2 classification relied on nephrologist review of historical records. Admission NPS combined albumin, total cholesterol, neutrophil-to-lymphocyte ratio, and lymphocyte-to-monocyte ratio. NPS was analyzed primarily as a continuous score using univariable and multivariable Firth penalized logistic regression with profile penalized-likelihood confidence intervals. Low (0–2) versus high (3–4) NPS comparisons were exploratory; no patient scored 0. **Results:** Overall, 26 patients (25.7%) died. Mortality was higher in the high-NPS than the low-NPS group (24/52 [46.2%] versus 2/49 [4.1%]; *p* < 0.001). After adjustment for age, New York Heart Association (NYHA) functional class, and left ventricular ejection fraction (LVEF), the odds ratio per one-point NPS increase was 3.42 (95% confidence interval [CI], 1.76–7.77; *p* < 0.001). The area under the receiver operating characteristic curve was 0.825 (percentile bootstrap 95% CI, 0.744–0.896). At the established NPS ≥ 3 category boundary, sensitivity was 92.3% (95% CI, 75.9–97.9%) and specificity was 62.7% (95% CI, 51.4–72.7%). Performance was estimated within the same cohort without optimism correction or external validation. **Conclusions:** Higher admission NPS was associated with in-hospital mortality after adjustment for age, NYHA class, and LVEF. These exploratory findings require external validation and assessment of incremental predictive value and clinical utility.

## 1. Introduction

Cardiorenal syndrome (CRS) encompasses a group of disorders in which acute or chronic dysfunction of the heart and kidneys may adversely affect the function of the other organ. The Acute Dialysis Quality Initiative classified CRS into five subtypes according to the primarily affected organ and the acute or chronic nature of the disease process [1]. Type 2 cardiorenal syndrome (CRS-2) specifically refers to kidney injury or dysfunction that develops or progresses as a consequence of chronic cardiac dysfunction [1,2].

The pathophysiology of CRS-2 extends beyond reduced cardiac output and impaired renal perfusion. Persistent venous congestion, activation of the renin–angiotensin–aldosterone and sympathetic nervous systems, systemic inflammation, and oxidative stress may all contribute to progressive renal injury [2,3]. Malnutrition is also common in chronic heart failure and is associated with hospitalization and mortality [4]. These observations support investigating the association of routine laboratory measures related to inflammatory and nutritional status with adverse outcomes in patients with chronic cardiac and renal dysfunction.

The Naples Prognostic Score (NPS) is a composite index derived from serum albumin, total cholesterol, neutrophil-to-lymphocyte ratio, and lymphocyte-to-monocyte ratio. It was originally developed to estimate long-term outcomes in patients undergoing surgery for colorectal cancer [5]. NPS has subsequently been evaluated in cardiovascular populations as a composite laboratory score whose components may reflect multiple aspects of systemic illness. Higher NPS values have been associated with increased in-hospital and follow-up mortality in patients with decompensated heart failure, as well as with long-term mortality across different heart failure phenotypes [6,7].

Despite these findings, evidence regarding the association between NPS and in-hospital mortality specifically in patients with CRS-2 remains limited. Accordingly, the present study aimed to evaluate the association between admission NPS and in-hospital mortality and to assess its within-cohort discriminatory performance in patients hospitalized for decompensated heart failure with clinical features consistent with CRS-2.

## 2. Materials and Methods

This retrospective observational cohort study was conducted in the Department of Cardiology, Sakarya University Training and Research Hospital, Sakarya, Türkiye, between 1 June 2021, and 1 August 2024. All heart failure admissions to the cardiology department during the study period were retrospectively screened for eligibility using the hospital records. The principal reason for the index hospitalization was decompensated heart failure in all patients included in the final cohort. The study protocol was approved by the local ethics committee (approval date: 19 September 2024; approval no. 05) and was conducted in accordance with the principles of the Declaration of Helsinki.

Patients were eligible for inclusion if they were aged ≥18 years, had documented chronic heart failure and persistent renal dysfunction, and had clinical features consistent with type 2 cardiorenal syndrome (CRS-2). Chronic heart failure was ascertained through review of previous discharge summaries and echocardiographic reports. New York Heart Association (NYHA) functional class was determined from the clinical assessment documented at initial hospital presentation and reflected the patient’s symptoms and functional limitation during the acute decompensated heart failure episode. It did not represent a separate assessment of usual pre-admission functional status.

Historical serum creatinine and estimated glomerular filtration rate (eGFR) measurements were reviewed to establish the presence and chronicity of renal dysfunction. At least two renal function measurements were reviewed for each patient. For this study, chronic kidney disease (CKD) was identified by an eGFR < 60 mL/min/1.73 m^2^ persisting for at least 3 months, consistent with the reduced-GFR criterion in the Kidney Disease: Improving Global Outcomes (KDIGO) definition of CKD. Classification was therefore not based on a single abnormal measurement or an isolated deterioration in renal function during the index hospitalization.

The study nephrologist retrospectively assigned the cardiorenal syndrome subtype using the available clinical records, previous discharge summaries, echocardiographic reports, and historical renal function measurements. Patients were classified as having features consistent with CRS-2 when the documented chronology supported chronic heart failure preceding or contributing to persistent renal dysfunction. This clinical assessment did not establish a definitive causal relationship between cardiac and renal disease.

The patient list was established before extraction of laboratory and in-hospital outcome data for the analytical dataset and before calculation of NPS. During CRS subtype classification, the study nephrologist was unaware of the patients’ in-hospital clinical course and outcomes, including survival status. NPS had not yet been calculated for the study. Historical clinical information required to determine eligibility and assess the cardiorenal relationship was reviewed during cohort identification.

Demographic, clinical, echocardiographic, and laboratory data were retrospectively obtained from the hospital’s electronic medical records. The variables collected included age, sex, diabetes mellitus, hypertension, atrial fibrillation, NYHA functional class, left ventricular ejection fraction (LVEF), admission hemoglobin, serum creatinine, eGFR, serum albumin, total cholesterol, neutrophil count, lymphocyte count, and monocyte count. Length of hospital stay, in-hospital mortality, and planned maintenance hemodialysis at discharge were also recorded.

Patients were excluded if they were younger than 18 years old; did not have documented chronic heart failure; had insufficient previous renal function measurements to establish persistent or progressive renal dysfunction during the course of chronic cardiac disease; or had an isolated or transient deterioration in renal function during the index hospitalization without prior evidence of chronic or progressive renal impairment. Active infection at admission was assessed retrospectively by reviewing the physical examination findings together with the available laboratory results documented in the medical records. Patients considered to have active infection on this clinical assessment were excluded because acute infection may substantially affect the inflammatory components of NPS. Patients whose clinical chronology did not support CRS-2 or was more consistent with another cardiorenal syndrome subtype were excluded. In addition, patients with missing laboratory data required for NPS calculation or missing in-hospital outcome data were excluded. Repeated admissions were accounted for before determining the number of unique patients screened. For patients with more than one eligible hospitalization, only the first eligible admission was included.

Baseline laboratory measurements, including hemoglobin and all four NPS components, were obtained from samples collected concurrently at initial hospital presentation. The analytical dataset did not include the timing of specific treatments relative to blood sampling. For additional descriptive characterization of cardiac function, recorded LVEF values were grouped as ≤40%, 41–49%, and ≥50%; these were reported as LVEF categories without separate adjudication of heart failure phenotypes. Laboratory-reported eGFR values, calculated using the CKD-EPI equation employed by the hospital laboratory, were used without recalculation. In the final analytical cohort, no missing values were present for the variables included in the primary analyses.

The Naples Prognostic Score (NPS) was calculated using admission laboratory measurements according to the published scoring criteria [5]. One point was assigned for each of the following findings: serum albumin < 4.0 g/dL, total cholesterol ≤ 180 mg/dL, neutrophil-to-lymphocyte ratio (NLR) ≥ 2.96, and lymphocyte-to-monocyte ratio (LMR) ≤ 4.44. The possible NPS range was 0–4, with higher scores indicating a greater number of components meeting the specified scoring criteria.

NPS was analyzed primarily as a continuous predictor, with the odds ratio estimated per one-point increase over the observed score range of 1–4. This specification assumes a linear association between NPS and the log odds of in-hospital mortality, conditional on the adjustment variables. For secondary exploratory analyses, patients were categorized as having low NPS (0–2) or high NPS (3–4). This boundary corresponds to the established NPS categories of 0, 1–2, and 3–4 reported in previous heart failure research [7], with the first two categories combined. Because no patient in the present cohort had an NPS of 0, the observed categorical comparison was between scores of 1–2 and 3–4.

The primary outcome was all-cause in-hospital mortality. The secondary exploratory outcome was planned maintenance hemodialysis at discharge, defined as hemodialysis initiated for the first time during the index hospitalization with a documented plan for maintenance hemodialysis after discharge. None of the included patients had received dialysis before the index admission. This endpoint was assessed only among patients discharged alive. The designation reflected the discharge treatment plan and did not establish permanent or long-term dialysis dependence, which was not confirmed through post-discharge follow-up. The comparison between NPS groups was unadjusted.

## 3. Statistical Analysis

Continuous variables were assessed for normality using the Shapiro–Wilk test. Normally distributed variables are presented as mean ± standard deviation and were compared using Welch’s *t*-test. Non-normally distributed variables are presented as median with interquartile range and were compared using the Mann–Whitney *U* test. Categorical variables are presented as numbers and percentages and were compared using Pearson’s chi-square test or Fisher’s exact test, as appropriate. The overall distribution of LVEF categories was compared using the Fisher–Freeman–Halton exact test because of sparse cell counts. Between-group hypothesis tests for serum albumin, total cholesterol, NLR, and LMR were not performed when comparing the low- and high-NPS groups because these components directly determined group membership.

In-hospital mortality was compared between NPS groups using Fisher’s exact test. Patient counts and mortality proportions were also summarized for each observed NPS value. The secondary exploratory comparison of planned maintenance hemodialysis at discharge was restricted to patients discharged alive and was performed using Fisher’s exact test without covariate adjustment. Patients who died during hospitalization were excluded from this discharge-outcome comparison. Length of hospital stay was compared between NPS groups using the Mann–Whitney *U* test in the full cohort, including patients who died during hospitalization.

Associations with in-hospital mortality were examined using Firth penalized logistic regression for both univariable and multivariable analyses. The same estimation approach was applied throughout because the cohort included 26 deaths and sparse events in some exposure categories, with the aim of reducing small-sample and separation-related estimation bias. In the primary adjusted model, NPS was entered as a continuous predictor and adjusted for age, NYHA functional class, and left ventricular ejection fraction (LVEF). These covariates were selected on clinical grounds rather than according to their statistical significance in univariable analyses. Age was included to account for age-related differences in prognosis, NYHA functional class to represent symptomatic and functional severity, and LVEF to characterize left ventricular systolic function. Given the 26 in-hospital deaths, the primary model was restricted to NPS and these three adjustment variables to limit model complexity. Odds ratios were reported per one-point increase in NPS, per 10-year increase in age, per one-class increase in NYHA functional class, and per 5 percentage-point increase in LVEF.

A secondary exploratory Firth regression model compared high NPS (3–4) with low NPS (0–2), adjusting for the same covariates as the primary continuous NPS model. Given the clinical importance of renal function in cardiorenal syndrome, admission eGFR was added to the primary model in an exploratory sensitivity analysis, with its odds ratio expressed per 10 mL/min/1.73 m^2^ increase. This analysis assessed the NPS association after additional adjustment for renal function at presentation. The distinction between the primary and exploratory models reflected the decision to retain a limited primary adjustment set in a cohort with few deaths; it was not based on the univariable *p*-value for eGFR. Admission eGFR was not considered a substitute for pre-admission renal function or the preceding renal trajectory.

NPS, age, and LVEF were entered as single linear terms in the models in which they were included; admission eGFR was similarly modeled in the exploratory extension. NYHA functional class was entered as an ordinal numeric variable using one model parameter. These specifications assumed a linear relationship between each predictor and the log odds of in-hospital mortality, including an equal change in log odds for each one-class increase in NYHA. Formal assessments using restricted cubic splines or categorical coding of NYHA class were not performed. These simplifying assumptions were retained to limit model complexity given the small number of deaths.

The discriminatory performance of NPS for in-hospital mortality was evaluated using receiver operating characteristic (ROC) analysis. The area under the ROC curve (AUC) was calculated in the original cohort. Its 95% confidence interval was estimated using 5000 patient-level bootstrap resamples of size 101 drawn with replacement (random seed, 42). Resampling was not stratified by outcome. The 2.5th and 97.5th percentiles of the bootstrap AUC distribution were used to obtain the percentile confidence interval. Bootstrap resampling quantified uncertainty around the AUC; it was not used for optimism correction. Sensitivity, specificity, positive predictive value, and negative predictive value were calculated at the NPS ≥ 3 boundary corresponding to the established high-NPS category, with 95% Wilson score confidence intervals without continuity correction. An exploratory Youden-index analysis assessed whether the same boundary maximized the index within this cohort. All performance measures were estimated in the same study population, without external validation.

Additional exploratory analyses examined the individual NPS components. The number of patients receiving each component point and mortality according to point status were reported. Separate univariable Firth logistic regression models compared patients receiving one point with those receiving zero points for each component, using profile penalized-likelihood confidence intervals and penalized-likelihood ratio tests. These analyses were not adjusted for clinical covariates or multiple testing.

The discrimination of the full NPS was compared with NLR as a continuous ratio and an inflammatory subscore defined as the sum of the NLR ≥ 2.96 and LMR ≤ 4.44 points, ranging from 0 to 2. We calculated 95% confidence intervals for AUCs and paired AUC differences using 5000 unstratified patient-level bootstrap resamples, with identical patient indices used for all scores within each resample. These analyses evaluated apparent discrimination without score recalibration, optimism correction, or external validation.

As an exploratory analysis, discrimination was compared between two Firth penalized logistic regression models: a clinical model containing age, NYHA functional class, and LVEF, and the same model with continuous NPS added. Both models were fitted and evaluated in the same 101 patients. The model-based AUCs and their difference, calculated as the AUC of the clinical model with NPS minus that of the clinical model without NPS, were reported descriptively, without formal hypothesis testing of the AUC difference. These estimates represent apparent discrimination; no optimism correction or external validation was performed for this comparison.

All tests were two-sided, and *p* < 0.05 was used as the nominal significance threshold. Exploratory analyses were not adjusted for multiple testing. Analyses were performed using Python 3.12.14. Firth regression was implemented in the custom module study_firth.py (version 1.0.0), using NumPy 2.3.5 and SciPy 1.17.0. The fit_firth function maximized the binomial log likelihood plus the Jeffreys penalty using scipy.optimize.minimize with the BFGS algorithm. All reported odds ratios were accompanied by 95% profile penalized-likelihood confidence intervals; *p*-values were calculated using penalized-likelihood ratio tests with a one-degree-of-freedom chi-square reference distribution. The profile_inference function reoptimized nuisance parameters at each fixed coefficient value and located confidence limits using scipy.optimize.brentq. The determinant of the full model information matrix was retained in the penalty during constrained fits.

## 4. Results

After repeated admissions had been accounted for, the medical records of 2150 unique patients were retrospectively screened. Renal dysfunction was identified in 960 patients, including 450 with chronic kidney disease. Among these 450 patients, 127 were considered potentially compatible with CRS-2 based on their historical clinical records and renal function trajectory and underwent detailed eligibility assessment. Following application of the inclusion and exclusion criteria, 26 patients were excluded, leaving 101 patients in the final analytical cohort. The patient selection process is summarized in Figure 1.

All included patients were hospitalized for decompensated heart failure. The observed NPS values ranged from 1 to 4; no patient had an NPS of 0. Of the study population, 49 patients (48.5%) were classified as having low NPS (0–2), whereas 52 patients (51.5%) had high NPS (3–4). Baseline demographic and clinical characteristics are presented in Table 1. No active malignancy or pre-existing liver disease was documented in the reviewed clinical records of the included patients.

The median age was 73 years (interquartile range [IQR], 64–81) in the overall cohort and did not differ significantly between the high- and low-NPS groups (74 (64–83) vs. 72 (63–77) years; *p* = 0.274). Admission serum creatinine did not differ significantly between the high- and low-NPS groups (2.22 [1.71–3.51] vs. 2.05 [1.56–2.64] mg/dL; *p* = 0.205). Median admission eGFR was 22.16 [15.18–35.12] mL/min/1.73 m^2^ in the high-NPS group and 30.80 [18.00–39.40] mL/min/1.73 m^2^ in the low-NPS group; the between-group difference was not statistically significant (*p* = 0.066). Mean admission hemoglobin was 10.33 ± 1.90 g/dL in the overall cohort, with no statistically significant difference between the high- and low-NPS groups (10.47 ± 1.83 vs. 10.18 ± 1.97 g/dL; *p* = 0.441).

Median LVEF was numerically lower in the high-NPS group than in the low-NPS group (30% (25–40) vs. 40% (30–45)); however, this difference was not statistically significant (*p* = 0.072). Overall, 70 patients (69.3%) had an LVEF ≤ 40%, 8 (7.9%) had an LVEF of 41–49%, and 23 (22.8%) had an LVEF ≥ 50%. The corresponding distributions by NPS group are presented in Table 1. The overall comparison of LVEF category distributions yielded *p* = 0.0496 using the Fisher–Freeman–Halton exact test. The distribution of NYHA functional class differed between the groups (overall *p* = 0.006), with class IV recorded in 30.8% of patients in the high-NPS group and 6.1% in the low-NPS group.

No statistically significant differences between the high- and low-NPS groups were observed in sex, diabetes mellitus, hypertension, or atrial fibrillation. The NPS components were summarized descriptively across these groups without hypothesis testing because they directly determined group membership. The high-NPS group had a higher median NLR and a lower median LMR than the low-NPS group.

Clinical outcomes are summarized in Table 2. Overall, 26 of the 101 patients (25.7%) died during hospitalization. In-hospital mortality was substantially higher in the high-NPS group than in the low-NPS group (24/52 [46.2%] vs. 2/49 [4.1%]; *p* < 0.001). The distribution of patients and in-hospital mortality by individual NPS is presented in Table 2, Panel B. NPS scores of 1, 2, 3, and 4 were observed in 15, 34, 22, and 30 patients, respectively. The corresponding numbers of deaths were 0, 2, 7, and 17, yielding mortality rates of 0.0%, 5.9%, 31.8%, and 56.7%. Thus, observed mortality increased progressively across the NPS values represented in this cohort.

A total of 75 patients survived to discharge. No patient in the cohort had received dialysis before the index admission. Maintenance hemodialysis was planned at discharge for 12 survivors, all of whom were in the high-NPS group and had started hemodialysis for the first time during the index hospitalization. This discharge plan was documented in 12 of 28 survivors in the high-NPS group (42.9%) and none of the 47 survivors in the low-NPS group (*p* < 0.001). This unadjusted secondary exploratory comparison was restricted to patients discharged alive and reflected the treatment plan at discharge, without confirmation of long-term dialysis dependence.

The median length of hospital stay was 9 days (IQR, 6–16) in the overall cohort, 8 days (IQR, 6–13) in the low-NPS group, and 10.5 days (IQR, 6–18) in the high-NPS group. The between-group difference was not statistically significant (*p* = 0.199).

The results of the univariable and multivariable Firth penalized logistic regression analyses for in-hospital mortality are presented in Table 3. In univariable analysis, each one-point increase in NPS was associated with higher odds of in-hospital mortality (OR, 4.15; 95% CI, 2.32–8.36; *p* < 0.001). When NPS was evaluated categorically, high NPS was associated with higher odds of mortality compared with low NPS (OR, 16.33; 95% CI, 4.81–85.19; *p* < 0.001). Age and NYHA functional class were also associated with mortality in the univariable Firth analyses. The OR was 2.52 for each 10-year increase in age (95% CI, 1.53–4.59; *p* < 0.001) and 5.82 for each one-class increase in NYHA functional class (95% CI, 2.75–14.09; *p* < 0.001). LVEF, admission creatinine, admission eGFR, sex, diabetes mellitus, hypertension, and atrial fibrillation were not significantly associated with in-hospital mortality in these analyses.

In the primary Firth penalized logistic regression model adjusted for age, NYHA functional class, and LVEF, each one-point increase in NPS was associated with higher odds of in-hospital mortality (OR, 3.42; 95% CI, 1.76–7.77; *p* < 0.001). Older age (per 10-year increase: OR, 2.42; 95% CI, 1.29–5.27; *p* = 0.004) and higher NYHA functional class (per one-class increase: OR, 4.46; 95% CI, 1.89–12.51; *p* < 0.001) were also associated with higher odds of in-hospital mortality in this model. The association with LVEF was not statistically significant (per increase of 5 percentage points: OR, 0.96; 95% CI, 0.73–1.26; *p* = 0.772).

In the secondary exploratory categorical model, high NPS remained associated with higher odds of in-hospital mortality after adjustment for the same covariates (OR, 11.63; 95% CI, 3.01–65.79; *p* < 0.001). Only two deaths occurred among the 49 patients in the low-NPS group, and the confidence interval for this estimate was wide. In the exploratory extension of the primary continuous NPS model that additionally included admission eGFR, the NPS association remained (per one-point increase: OR, 3.54; 95% CI, 1.81–8.07; *p* < 0.001). The association with admission eGFR was not statistically significant (per 10 mL/min/1.73 m^2^ increase: OR, 1.29; 95% CI, 0.77–2.14; *p* = 0.330).

The albumin, cholesterol, NLR, and LMR component points were assigned to 92 (91.1%), 78 (77.2%), 45 (44.6%), and 54 (53.5%) patients, respectively. In separate exploratory univariable Firth analyses, the NLR point (OR, 24.84; 95% CI, 7.23–130.74; *p* < 0.001) and LMR point (OR, 14.62; 95% CI, 4.32–76.14; *p* < 0.001) were associated with in-hospital mortality. The associations for the albumin point (OR, 2.14; 95% CI, 0.45–20.82; *p* = 0.371) and cholesterol point (OR, 0.94; 95% CI, 0.35–2.80; *p* = 0.912) were not statistically significant. Component frequencies, mortality according to point status, and effect estimates are presented in Supplementary Table S1.

The discriminatory performance of NPS for in-hospital mortality is presented in Table 4 and Figure 2. Within this cohort, NPS demonstrated good discrimination, with an AUC of 0.825 (percentile bootstrap 95% CI, 0.744–0.896). At the NPS ≥ 3 boundary corresponding to the high-NPS category, sensitivity was 92.3% (95% CI, 75.9–97.9%) and specificity was 62.7% (95% CI, 51.4–72.7%). The positive predictive value was 46.2% (24/52; 95% CI, 33.3–59.5%), and the negative predictive value was 95.9% (47/49; 95% CI, 86.3–98.9%). These predictive values were estimated in a cohort with an in-hospital mortality prevalence of 25.7%. Two of the 49 patients with NPS < 3 died during hospitalization. The exploratory Youden-index analysis identified the same category boundary as maximizing the index within this cohort. This concordance does not constitute independent validation. All performance estimates were derived from the same 101 patients, without optimism correction or external validation.

In exploratory comparisons, the AUC was 0.861 (bootstrap 95% CI, 0.761–0.940) for NLR alone and 0.828 (bootstrap 95% CI, 0.746–0.901) for the inflammatory subscore, compared with 0.825 (bootstrap 95% CI, 0.744–0.896) for the full NPS. The paired AUC difference was −0.036 for full NPS minus NLR (95% CI, −0.107 to 0.033) and −0.004 for full NPS minus the inflammatory subscore (95% CI, −0.051 to 0.039). Both difference intervals included zero. These within-cohort comparisons did not demonstrate additional discrimination for the full NPS and do not establish equivalence or superiority between the scores (Supplementary Table S2).

In an exploratory comparison, the clinical model containing age, NYHA functional class, and LVEF had an apparent AUC of 0.869. The corresponding model with continuous NPS added had an apparent AUC of 0.920, yielding an AUC difference of 0.051. This difference was reported descriptively without formal hypothesis testing. Both models were fitted and evaluated in the same 101 patients, without optimism correction or external validation.

## 5. Discussion

In this retrospective cohort of patients hospitalized for decompensated heart failure with clinical features consistent with CRS-2, higher admission NPS was associated with in-hospital mortality after adjustment for age, NYHA functional class, and LVEF. The association was also observed in the exploratory model that additionally included admission eGFR. NPS showed apparent discrimination within this cohort, while the component analyses did not demonstrate additional discrimination of the full score over NLR alone or the inflammatory subscore. These findings support further investigation of NPS as a prognostic marker, but do not establish validated clinical risk prediction or a specific nutritional–inflammatory mechanism.

Type 2 cardiorenal syndrome is a heterogeneous clinical condition arising from sustained interactions between chronic cardiac dysfunction and progressive renal impairment. Its pathophysiology extends beyond reduced cardiac output or renal hypoperfusion and involves persistent venous congestion, neurohormonal activation, endothelial dysfunction, oxidative stress, and systemic inflammation (1–3). These mechanisms may reinforce one another, leading to progressive deterioration in both cardiac and renal function. In patients with advanced decompensated heart failure, Mullens et al. found that higher central venous pressure was associated with worsening renal function [8]. This observation supports the relevance of venous congestion when interpreting cardiorenal dysfunction.

Nutritional abnormalities may accompany chronic heart failure and coexist with renal dysfunction. In chronic heart failure, reduced appetite, intestinal congestion, impaired nutrient absorption, systemic inflammation, increased catabolic activity, and loss of muscle mass may all contribute to malnutrition. Malnutrition has consistently been associated with hospitalization and mortality in patients with heart failure [4]. The adverse consequences may be more pronounced when renal dysfunction is also present. Otaki et al. demonstrated a close relationship between malnutrition and renal dysfunction and reported that their coexistence was associated with adverse clinical outcomes in patients with heart failure [9]. These observations support the concept that nutritional and renal abnormalities should not be considered independently in patients with cardiorenal disease.

Systemic inflammation provides another link between cardiac dysfunction, renal impairment, and poor clinical outcomes. Neutrophilia may reflect activation of innate inflammatory pathways, whereas relative lymphopenia may indicate physiological stress, impaired immune regulation, and a more advanced disease state. A systematic review and meta-analysis by Vakhshoori et al. showed that an elevated neutrophil-to-lymphocyte ratio was associated with adverse outcomes in heart failure populations [10]. Similarly, Ang et al. reported that higher NLR values were associated with increased mortality in patients with acute decompensated heart failure [11]. Although elevated NLR may accompany systemic inflammation, it is also influenced by physiological stress and underlying illness and should not be interpreted as a disease-specific marker.

The Naples Prognostic Score incorporates serum albumin, total cholesterol, NLR, and LMR and was initially developed in patients undergoing surgery for colorectal cancer [5]. These components are influenced by multiple biological processes and should not be interpreted as specific measures of nutritional status or a single inflammatory pathway. The American Society for Parenteral and Enteral Nutrition (ASPEN) position paper emphasizes that inflammation and altered capillary permeability influence serum albumin concentrations; albumin should therefore not be interpreted as a direct measure of nutritional status [12]. Albumin and total cholesterol concentrations may be affected by nutritional disturbances, systemic inflammation, congestion, hepatic dysfunction, hemodilution, and advanced chronic illness. Similarly, NLR and LMR are nonspecific indicators of inflammatory and immune responses and physiological stress. Higher NPS values may therefore reflect greater overall disease burden and reduced physiological reserve. The present study cannot determine whether the observed association with mortality is specifically mediated by inflammation and malnutrition or reflects these broader influences.

The exploratory component analyses further qualify the interpretation of NPS in this cohort. Albumin and cholesterol points were assigned to most patients, limiting variation in these components at the scoring thresholds. The NLR and LMR points were associated with mortality in univariable analyses, whereas the albumin and cholesterol point associations were imprecisely estimated and were not statistically significant. The full NPS did not demonstrate additional apparent discrimination over NLR alone or the inflammatory subscore. These findings suggest that much of the observed discrimination was retained by the inflammatory measures, but do not establish superiority or equivalence between scores. Moreover, NLR and LMR both depend on lymphocyte count and may convey overlapping information. The present findings therefore do not establish a specifically combined nutritional and inflammatory mechanism, nor do they exclude a clinically relevant contribution of nutritional status.

Associations between NPS and adverse outcomes have been reported in several cardiovascular settings. Erdogan et al. reported associations with mortality and rehospitalization in patients hospitalized with decompensated heart failure [6], while Aydın et al. reported an association with long-term mortality in patients with heart failure [7]. Associations have also been reported in patients with ST-segment elevation myocardial infarction [13,14] and after transcatheter aortic valve replacement [15]. In a cohort of patients with CKD undergoing percutaneous coronary intervention, Zhang et al. found that higher NPS was associated with major adverse cardiac events [16]. More recently, Karaduru Avcı et al. reported an association between NPS and rapid 12-month kidney function decline in a multicenter cohort of patients with stage 3–4 CKD [17]. These studies extend the settings in which NPS has been investigated, although differences in patient characteristics and outcome definitions limit direct comparisons with our findings.

The present study extends the evidence on NPS to patients with clinical features consistent with CRS-2. The association between NPS and in-hospital mortality remained after adjustment for age, NYHA functional class, and LVEF, and was also observed in the exploratory model that additionally included admission eGFR. These findings describe associations conditional on the variables included in the models. They do not establish that NPS captures clinical severity beyond comprehensive routine assessment or provides validated incremental predictive value. Residual confounding by renal disease severity, congestion, comorbidities, and treatment remains possible.

Age and NYHA functional class remained important clinical indicators, which is consistent with their established roles in heart failure prognosis. The association between left ventricular ejection fraction and mortality was not statistically significant in the adjusted model. Ejection fraction alone may not adequately represent congestion, right ventricular dysfunction, cardiac output, valvular disease, systemic inflammation, or functional reserve. Furthermore, patients with preserved or mildly reduced ejection fraction may still have advanced heart failure and severe cardiorenal involvement.

The continuous NPS analysis constitutes the principal analysis because it retains more information than dichotomization. The categorical comparison should be interpreted as secondary and exploratory. Only two deaths occurred among the 49 patients in the low-NPS group, resulting in considerable uncertainty around the categorical effect estimate (adjusted OR, 11.63; 95% CI, 3.01–65.79). Although Firth penalization reduces small-sample and separation-related estimation bias [18], it does not eliminate the imprecision associated with sparse events. The conclusions therefore primarily reflect the continuous NPS analysis.

The progressive increase in observed mortality across NPS scores of 1–4 is consistent with a graded association between higher NPS and mortality. However, these descriptive proportions do not formally establish linearity of the adjusted association on the log-odds scale. The per-point odds ratio should therefore be interpreted as a model-based summary over the observed score range. The absence of deaths among the 15 patients with an NPS of 1 should not be interpreted as absence of risk, and the study provides no direct information on outcomes among patients with an NPS of 0.

The ROC analysis showed good discrimination of NPS within this cohort. The exploratory Youden-index analysis identified the same boundary as the established NPS ≥3 category, but this within-cohort agreement does not constitute independent validation. Although the negative predictive value was 95.9%, its 95% confidence interval ranged from 86.3% to 98.9%, and two deaths occurred among the 49 patients with NPS <3. The observed mortality in this group was 4.1% (95% CI, 1.1–13.7%), illustrating the uncertainty associated with the small number of events. These findings do not validate the use of NPS <3 to identify clinically low-risk patients. Furthermore, positive and negative predictive values depend on the underlying mortality prevalence, which was 25.7% in this cohort, and may differ in other populations. Bootstrap resampling quantified uncertainty around the AUC without correcting for optimism. External validation is required before this category boundary can inform clinical decisions.

The secondary dialysis finding should be regarded as exploratory. All 12 patients for whom maintenance hemodialysis was planned at discharge had initiated hemodialysis during the index hospitalization, and none had received dialysis previously. However, the endpoint reflected the discharge treatment plan and did not establish irreversible kidney failure or sustained dialysis dependence after discharge. The analysis was restricted to patients discharged alive and was therefore susceptible to survivor selection, with in-hospital death constituting a competing event that precluded assessment of the discharge endpoint. The comparison was also not adjusted for admission eGFR, pre-existing CKD severity, or the preceding renal trajectory. These limitations prevent interpretation of this finding as evidence that NPS predicts long-term dialysis dependence.

Although NPS is calculated from routine laboratory measurements, ease of calculation does not establish clinical utility. In the exploratory comparison of otherwise identical Firth models, adding continuous NPS to age, NYHA functional class, and LVEF increased the apparent AUC from 0.869 to 0.920, an absolute difference of 0.051. This comparison was descriptive and was not subjected to formal hypothesis testing. Both models were fitted and evaluated in the same 101 patients, with only 26 deaths, and the performance estimates were not corrected for optimism. Moreover, this limited clinical model does not represent comprehensive routine clinical assessment, and NT-proBNP was unavailable for analysis. The observed increase in apparent discrimination therefore does not establish validated incremental predictive value or improved clinical decision-making. Further evaluation should include external validation, assessment of calibration, and evaluation of clinical net benefit in the intended clinical setting [19].

This study has several limitations. Its retrospective, single-center design may limit generalizability. CRS-2 classification relied on the study nephrologist’s review of existing clinical records and historical renal measurements, which were not obtained at uniform intervals. Retrospective chronology cannot reliably establish the causal direction between cardiac and renal dysfunction or completely distinguish CRS-2 from CRS-4 or coexisting chronic heart and kidney disease. Contributions from diabetes, hypertension, and other renal disorders cannot be fully separated. Requiring sufficient historical clinical and renal information may also have introduced selection bias.

The primary model adjusted only for age, NYHA functional class, and LVEF; residual confounding by renal disease severity, comorbidities, congestion, and treatment therefore remains possible. Admission eGFR was included only in an exploratory model and did not characterize pre-admission CKD severity or the preceding renal trajectory. NT-proBNP, heart failure etiology, sodium, objective congestion measures, and detailed heart failure medication data were unavailable for analysis. Information on shock or intensive-care admission, systemic inflammatory disease, statin use, and corticosteroid use was also unavailable. Although all NPS components were measured from concurrent samples obtained at initial presentation, the timing of sampling relative to specific treatments could not be established.

The small number of deaths (*n* = 26), including only two among the 49 patients in the low-NPS group, limits estimate precision and model complexity. Firth penalization reduces small-sample estimation bias but does not eliminate residual confounding or overfitting. Continuous predictors were entered linearly on the log-odds scale, and NYHA class was modeled as a single ordinal term; these simplifying assumptions were not formally assessed. NYHA class reflected the acute condition at initial presentation rather than usual pre-admission functional status. No patient had an NPS of 0, limiting inference at that end of the score range.

All discrimination and threshold-performance estimates were obtained in the same cohort. Bootstrap confidence intervals quantified uncertainty without optimism correction, and external validation was not performed. Positive and negative predictive values depend on mortality prevalence and may differ in other populations. The exploratory clinical model comparison provided apparent AUC estimates and did not establish validated incremental predictive value. Calibration and clinical net benefit were not assessed.

The NPS components are nonspecific and cannot identify a particular inflammatory or nutritional mechanism. Component analyses were exploratory and were not adjusted for clinical covariates or multiple testing. Limited variation in the albumin and cholesterol point indicators, including only nine patients without the albumin point, reduced precision. The nonsignificant associations of these two indicators do not establish that nutritional factors are unrelated to mortality.

Finally, the unadjusted dialysis analysis was restricted to survivors and was susceptible to survivor selection and competing in-hospital death. The endpoint reflected a discharge treatment plan without confirmation of sustained dialysis dependence after discharge. The comparison did not account for admission eGFR, pre-existing CKD severity, or the preceding renal trajectory.

## 6. Conclusions

In this retrospective, single-center cohort of patients with clinical features consistent with CRS-2, the association between higher admission NPS and in-hospital mortality remained after adjustment for age, NYHA functional class, and LVEF. Although NPS demonstrated good discrimination within the cohort, residual confounding and uncertainty in cardiorenal syndrome classification limit interpretation. These exploratory findings do not establish validated incremental predictive value or clinical utility. Larger prospective multicenter studies with standardized clinical characterization, validation in independent populations, and assessment of calibration and clinical net benefit are needed before NPS can be recommended for clinical risk stratification.

## Figures and Tables

**Figure 1:**
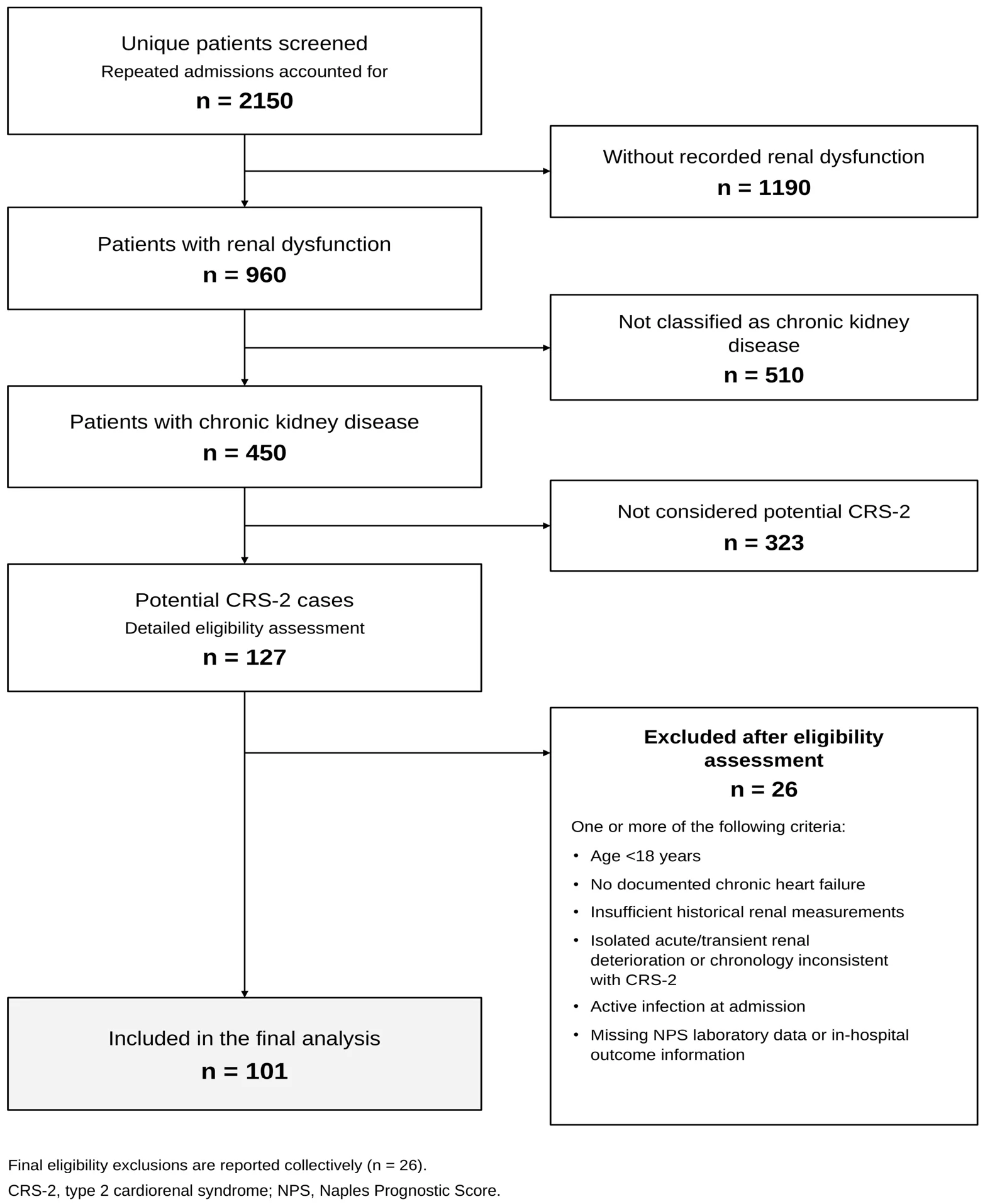
Patient selection flow diagram showing the screening stages, eligibility assessment, exclusions, and final analytical cohort.

**Figure 2:**
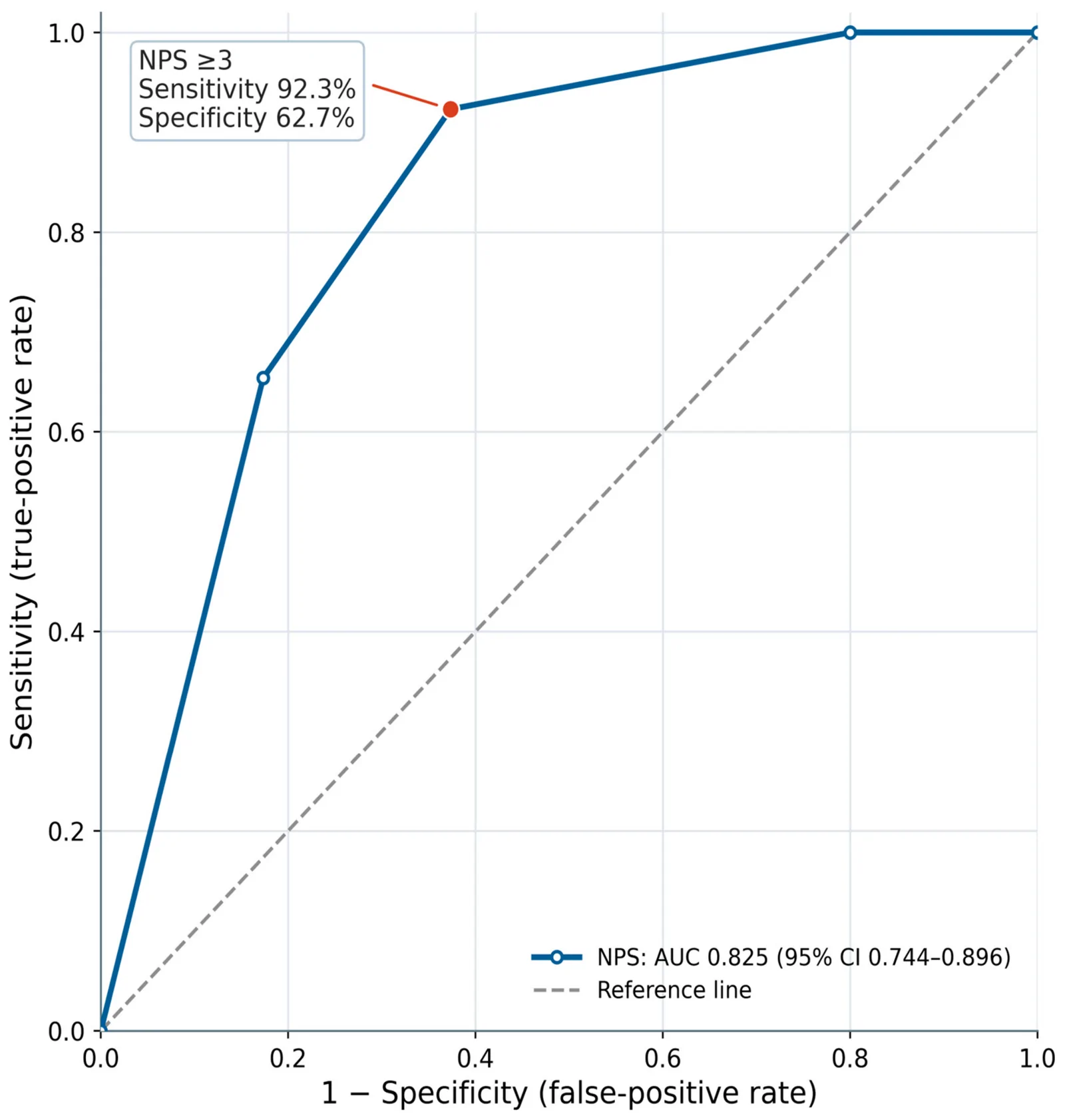
Receiver operating characteristic curve of the Naples Prognostic Score for predicting in-hospital mortality.

**Table 1:** Baseline Characteristics by NPS Group.

Variable	Overall (*n* = 101)	Low NPS 0–2 (*n* = 49)	High NPS 3–4 (*n* = 52)	*p*-Value
Age, years	73.00 [64.00–81.00]	72.00 [63.00–77.00]	74.00 [64.00–83.00]	0.274
Admission creatinine, mg/dL	2.10 [1.68–2.73]	2.05 [1.56–2.64]	2.22 [1.71–3.51]	0.205
Admission eGFR, mL/min/1.73 m^2^	25.00 [17.30–36.49]	30.80 [18.00–39.40]	22.16 [15.18–35.12]	0.066
LVEF, %	35.00 [30.00–45.00]	40.00 [30.00–45.00]	30.00 [25.00–40.00]	0.072
LVEF ≤ 40%, *n* (%)	70 (69.3%)	30 (61.2%)	40 (76.9%)	0.0496
LVEF 41–49%, *n* (%)	8 (7.9%)	7 (14.3%)	1 (1.9%)	
LVEF ≥ 50%, *n* (%)	23 (22.8%)	12 (24.5%)	11 (21.2%)	
Admission hemoglobin, g/dL	10.33 ± 1.90	10.18 ± 1.97	10.47 ± 1.83	0.441
Albumin, g/dL *	3.28 ± 0.55	3.31 ± 0.56	3.25 ± 0.54	—
Total cholesterol, mg/dL *	150.00 [114.00–178.00]	147.00 [114.00–182.00]	151.00 [121.75–172.00]	—
Neutrophil-to-lymphocyte ratio *	2.76 [2.11–5.32]	2.16 [1.77–2.37]	5.22 [3.58–9.17]	—
Lymphocyte-to-monocyte ratio *	3.74 [1.54–5.55]	5.55 [4.89–6.19]	1.64 [1.17–2.68]	—
Female sex, *n* (%)	54 (53.5%)	24 (49.0%)	30 (57.7%)	0.380
Diabetes mellitus, *n* (%)	56 (55.4%)	27 (55.1%)	29 (55.8%)	0.946
Hypertension, *n* (%)	85 (84.2%)	41 (83.7%)	44 (84.6%)	0.897
Atrial fibrillation, *n* (%)	34 (33.7%)	13 (26.5%)	21 (40.4%)	0.141
NYHA class 2, *n* (%)	37 (36.6%)	20 (40.8%)	17 (32.7%)	0.006
NYHA class 3, *n* (%)	45 (44.6%)	26 (53.1%)	19 (36.5%)	
NYHA class 4, *n* (%)	19 (18.8%)	3 (6.1%)	16 (30.8%)	

* Components of the Naples Prognostic Score (NPS). Between-group hypothesis tests were not performed for these variables because they were used to define the NPS groups.

**Table 2:** Clinical Outcomes by NPS Group and In-Hospital Mortality by Individual NPS: (A) Clinical outcomes by NPS group. (B) In-hospital mortality by individual NPS.

(A)					
Outcome		Population	Low NPS 0–2	High NPS 3–4	*p*-Value
In-hospital mortality, *n* (%)		All patients (*n* = 101)	2 (4.1%)	24 (46.2%)	<0.001
Planned maintenance hemodialysis at discharge (exploratory outcome), *n* (%)		Survivors (*n* = 75)	0/47 (0.0%)	12/28 (42.9%)	<0.001
Length of hospital stay, days, median [IQR]		All patients (*n* = 101)	8 (6–13)	10.5 (6–18)	0.199
(B)					
NPS	Patients, *n*	Deaths, *n*			In-Hospital Mortality, %
1	15	0			0.0
2	34	2			5.9
3	22	7			31.8
4	30	17			56.7
Total	101	26			25.7

**Table 3:** Firth penalized logistic regression analyses for in-hospital mortality. (A) Primary continuous NPS model, (B) secondary exploratory categorical NPS model.

(A)				
Variable	Univariable Firth OR (95% CI)	*p*-Value	Adjusted Firth OR (95% CI)	*p*-Value
NPS, per 1-point increase	4.15 (2.32–8.36)	<0.001	3.42 (1.76–7.77)	<0.001
Age, per 10-year increase	2.52 (1.53–4.59)	<0.001	2.42 (1.29–5.27)	0.004
NYHA class, per 1-class increase	5.82 (2.75–14.09)	<0.001	4.46 (1.89–12.51)	<0.001
LVEF, per 5 percentage-point increase	0.95 (0.78–1.14)	0.560	0.96 (0.73–1.26)	0.772
Admission creatinine, per 1 mg/dL increase	0.90 (0.57–1.34)	0.612	—	—
Admission eGFR, per 10 mL/min/1.73 m^2^ increase	0.90 (0.62–1.29)	0.586	—	—
Female vs. male sex	1.25 (0.52–3.07)	0.626	—	—
Diabetes mellitus	0.91 (0.38–2.23)	0.842	—	—
Hypertension	0.98 (0.32–3.54)	0.979	—	—
Atrial fibrillation	1.66 (0.66–4.10)	0.276	—	—
(B)				
High NPS (3–4) vs. low NPS (0–2)	16.33 (4.81–85.19)	<0.001	11.63 (3.01–65.79)	<0.001
Age, per 10-year increase	2.52 (1.53–4.59)	<0.001	2.53 (1.37–5.36)	0.002
NYHA class, per 1-class increase	5.82 (2.75–14.09)	<0.001	4.36 (1.84–12.33)	<0.001
LVEF, per 5 percentage-point increase	0.95 (0.78–1.14)	0.560	0.92 (0.71–1.19)	0.525

**Table 4:** Within-cohort discriminatory performance of NPS for in-hospital mortality.

AUC (95% CI)	NPS Category Boundary	Sensitivity (95% CI)	Specificity (95% CI)	PPV (95% CI)	NPV (95% CI)
0.825 (0.744–0.896)	NPS ≥ 3	92.3% (75.9–97.9%)	62.7% (51.4–72.7%)	46.2% (33.3–59.5%)	95.9% (86.3–98.9%)

## Data Availability

The datasets used and/or analyzed during the current study are available from the corresponding author on reasonable request.

## Supplementary Materials

The following supporting information can be downloaded at: https://www.mdpi.com/article/10.3390/diagnostics16193185/s1, Table S1. NPS component points and their associations with in-hospital mortality; Table S2. Exploratory comparison of discrimination for in-hospital mortality.
