# Supplementary material for: Association Between the Naples Prognostic Score and In-Hospital Mortality in Patients with Type 2 Cardiorenal Syndrome: A Retrospective Cohort Study
Source: Diagnostics (Basel). 2026 Sep 30;16(19):3185. doi: 10.3390/diagnostics16193185 (PMC13664215; doi:10.3390/diagnostics16193185)
Supplement: Supplementary file 1 [file diagnostics-16-03185-s001.zip › diagnostics-4539148-supplementary.pdf]

Supplementary Tables

Table S1. NPS component points and their associations with in-hospital mortality.

Criterion for 1 point	Patients receiving 1 point, n (%)	Mortality with 1 point, deaths/n (%)	Mortality with 0 points, deaths/n (%)	Univariable Firth OR (95% CI)	p-value
Albumin <4.0 g/dL	92 (91.1)	25/92 (27.2)	1/9 (11.1)	2.14 (0.45–20.82)	0.371
Total cholesterol ≤180 mg/dL	78 (77.2)	20/78 (25.6)	6/23 (26.1)	0.94 (0.35–2.80)	0.912
NLR ≥2.96	45 (44.6)	24/45 (53.3)	2/56 (3.6)	24.84 (7.23–130.74)	<0.001
LMR ≤4.44	54 (53.5)	24/54 (44.4)	2/47 (4.3)	14.62 (4.32–76.14)	<0.001

Table S2. Exploratory comparison of discrimination for in-hospital mortality.

Score	Apparent AUC	Bootstrap 95% CI
Full NPS	0.825	0.744–0.896
NLR as a continuous ratio	0.861	0.761–0.940
Inflammatory subscore (NLR and LMR points)	0.828	0.746–0.901

Paired differences in AUC

Comparison	AUC difference	Paired bootstrap 95% CI
Full NPS minus NLR	−0.036	−0.107 to 0.033
Full NPS minus inflammatory subscore	−0.004	−0.051 to 0.039
